# The Values of Systemic Immune-Inflammation Index and Neutrophil–Lymphocyte Ratio in Predicting Biochemical Recurrence in Patients With Localized Prostate Cancer After Radical Prostatectomy

**DOI:** 10.3389/fonc.2022.907625

**Published:** 2022-06-02

**Authors:** Shuo Wang, Xiao Yang, Ziyi Yu, Peng Du, Xinan Sheng, Yudong Cao, Xieqiao Yan, Jinchao Ma, Yong Yang

**Affiliations:** ^1^Key Laboratory of Carcinogenesis and Translational Research (Ministry of Education), Urological Department, Peking University Cancer Hospital & Institute, Beijing, China; ^2^Key Laboratory of Carcinogenesis and Translational Research (Ministry of Education), Department of Renal Cancer and Melanoma, Peking University Cancer Hospital & Institute, Beijing, China

**Keywords:** prostate cancer, systemic immune-inflammation index, neutrophil–lymphocyte ratio, inflammatory markers, biochemical recurrence

## Abstract

**Purpose:**

To investigate the association between preoperative systemic immune-inflammation index (SII) and neutrophil–lymphocyte ratio (NLR) and oncological outcomes in localized prostate cancer (PCa) patients after radical prostatectomy (RP).

**Methods:**

Between January 2014 and December 2019, 291 patients with pathologically confirmed localized PCa who underwent RP were included in this study. The threshold values of SII and NLR for biochemical recurrence (BCR) were calculated according to Youden’s index based on the receiver operating characteristic (ROC) curve, then the patients were divided into two groups by the threshold values of SII and NLR, and the clinicopathological outcomes were analyzed and compared between groups, respectively. The binary logistic regression model was used to evaluate the association between SII, NLR, and pathological outcomes including Gleason score (GS) and pathological T (pT) stage. Kaplan–Meier curves and univariable and multivariable Cox regression models were used to determine the association between high SII, high NLR, and BCR-free survival, respectively.

**Results:**

The median follow-up time was 48 months (IQR 36–62), and 114 (39.18%) patients developed BCR. The AUC of SII for BCR was 0.813 (*P* < 0.001), with a threshold value of 528.54, a sensitivity of 72.9%, and a specificity of 76.3%; the AUC of NLR for BCR was 0.824 (*P* < 0.001), with a threshold value of 2.62, a sensitivity of 71.2%, and a specificity of 81.6%. Patients were divided into two groups according to the threshold values of SII and NLR, respectively. Patients in the high SII group had higher tPSA, GS, pT stage, and BCR rate than patients in the low SII group (*P* = 0.004, 0.04, 0.007, and <0.001, respectively), and patients in the high NLR group had higher tPSA, GS, pT stage, and BCR rate than patients in the low NLR group (*P* = 0.04, 0.02, 0.006, and <0.001, respectively). Multivariable logistic regression analysis revealed that high SII was significantly correlated with adverse pathological outcomes of GS (HR, 1.656; 95% CI, 1.00–2.742, *P* = 0.042) and pT stage (HR, 1.478; 95% CI, 0.972–3.64, *P* = 0.028); there was no association between high NLR and pathological events. Kaplan–Meier analysis showed significantly poorer BCR-free survival in patients with high SII or high NLR (*P* < 0.001 and <0.001, respectively). By using the multivariable Cox regression model, high SII (HR, 4.521; 95% CI, 2.262–9.037, *P* < 0.001) and high NLR (HR, 4.787; 95% CI, 2.339–9.798, *P* < 0.001) were both significant predictors of BCR after RP.

**Conclusion:**

High SII was significantly related to unfavorable clinicopathological outcomes. High preoperative SII and NLR were related to higher BCR rate in localized PCa after RP, and they were all independent risk factors associated with shorter BCR-free survival. These two factors might provide promising and inexpensive methods for predicting clinical outcomes in patients with RP.

## Introduction

For localized prostate cancer (PCa), the most useful treatment method is radical prostatectomy. Unfortunately, approximately 30%–50% of patients will experience biochemical recurrence (BCR) after radical prostatectomy (RP), which is closely associated with tumor recurrence and metastasis (1). Many factors may influence the prostate-specific antigen (PSA) level after RP, and a retrospective study demonstrated that smoking status might be one of the most important factors (2). Meanwhile, several factors including Gleason score (GS) and clinical or pathological stage may be used for predicting BCR after RP as proven by several studies, but they lack the accuracy to guide the following therapeutic approach (2, 3). Therefore, reliable, easily accessible, and inexpensive markers are needed for assessing clinical outcomes in patients with localized PCa after RP.

The association between inflammation and PCa has been proven by literature (4). Neutrophil–lymphocyte ratio (NLR) is a well-known inexpensive and effective representative marker of an inflammatory condition. It has been proven to be positively associated with prognosis in various kinds of malignant tumors (5, 6). Regarding PCa, NLR was revealed to be an independent predictor for overall survival (OS) in patients with metastatic castration-resistant prostate cancer (mCRPC) (7). Recently, one retrospective study has shown a significantly worse prognosis in metastasis-free and OS of localized PCa patients with high NLR after radiotherapy (8). However, there is a paucity of studies about the association between NLR and clinical and pathological outcomes in localized PCa after RP.

In addition, another novel inflammatory marker, systemic immune-inflammation index (SII) which combines components of NLR and platelet–lymphocyte ratio (PLR), has been proven to be a more powerful method of predicting occurrence and progression in several kinds of tumors (9–11). In terms of PCa, it was firstly described in 2016 and was considered a powerful marker for predicting the prognosis of mCRPC (12). However, there are no data on the predictive value of SII on BCR in the setting of localized PCa after RP.

Thus, in this retrospective study, we aimed to evaluate the values of preoperative NLR and SII in predicting BCR after RP and detected their association with clinicopathological outcomes.

## Material and Methods

This retrospective study was carried out at Peking University Cancer Hospital & Institute and got the approval of the Medical Ethics Review Committee of Peking University Cancer Hospital & Institute (protocol code 2020KT30).

### Patients

Two hundred and ninety-one patients with localized PCa who underwent RP, consisting of 287 with laparoscopic RP and 4 with open RP, between January 2014 and December 2019z were reviewed. Among these patients, no one received neoadjuvant therapy before RP and adjuvant therapy after RP until the detection of BCR. In patients with smoking status, smoking was recommended to be ceased 1 month before RP. Preoperative clinical characteristics including age, serum total PSA (tPSA) value, total prostate volume (TPV), body mass index (BMI), and complete blood count (CBC)-based parameters as well as postoperative pathological and BCR outcomes were collected and compared according to the level of NLR and SII, respectively. Data of risk factors related to BCR including GS, pT stage, NLR, and SII were collected, and their associations with BCR-free survival time were analyzed. A single preoperative CBC with differential was performed as part of the routine assessment testing 1–2 days before RP simultaneously with the tPSA value. The CBC-based parameters including NLR and SII were used in this study. To ensure the CBCs were not affected by other factors, patients who met one of the following criteria were excluded: any surgical intervention within 1 month, non-steroidal anti-inflammatory drugs used within 1 month, acute or chronic infection, malignant tumors in other organs, and systemic inflammatory disease.

### Procedure

Ultrasound-guided 13-core transrectal prostate biopsy was performed in patients with PSA >4 ng/ml at our institute. The results of serum tPSA value and CBC-based parameters were collected just 1–2 days before the RP surgery and at least 3 weeks after the prostate biopsy to minimize the effect of the prostate biopsy. MRI, emission computed tomography (ECT), or CT was performed before surgery to confirm no bone, lymph node, or distant organ metastasis. Laparoscopic RP or open RP was performed in patients with PCa at least 30 days after the biopsy. Extrafascial radical prostatectomy through an extraperitoneal approach was performed by skilled and experienced surgeons in our institute according to the technique of Walsh et al., and standard pelvic lymph node dissection was performed in all patients (13). All specimens were assessed by a sophisticated pathologist at our institute, and serum tPSA value was detected every 1–3 months after RP.

### Variables

The prostate was measured in 3-dimensional aspects, and its volume was estimated with the modified ellipsoid formulation in cm^3^ (0.523 [length × width × height]) after surgery. Pathologic GSs were recorded and patients were staged according to the 2010 American Joint Committee on Cancer system (AJCC, pathologic stages T1–T4) (14). Tumors were classified into low (GS ≤ 6), intermediate (GS = 7), and high grade (GS ≥ 8) according to the D’Amico risk classification (15). NLR and SII were calculated by using the numbers of blood cell count-based systemic markers of inflammation. The NLR and SII were calculated as follows: NLR = neutrophil count/lymphocyte count; SII = platelet count × neutrophil count/lymphocyte count. SII was presented as a combination of NLR and PLR (9, 16). Body mass index (BMI) = weight (kg)/height (meter)^2^. BCR was defined as at least two consecutive serum tPSA ≥0.2 ng/ml according to the guidelines of the American Urological Association (17), and data of time free from BCR were collected.

### Statistical Analysis

Measurement data confirming normal distribution analyzed by the Shapiro–Wilk test are presented as mean ± SD. The independent sample *t*-test was used to evaluate the differences between continuous variables, while chi-square tests were performed to examine categorical variables. To determine the optimal cutoff value of NLR and SII for BCR, Youden’s index was calculated using the receiver operating characteristic curve (ROC), and the corresponding specificity–sensitivity levels were provided. Youden’s index was defined as YI_(C)_ = max c [Se_(C)_ + SP_(C)_ − 1]. The binary logistic regression model (univariate and multivariate analysis) was used to evaluate the association between NLR, SII, and adverse pathological events, which were all compared with the reference group (Ref). Kaplan–Meier analyses were performed for BCR-free survival according to NLR and SII using the log-rank test, and the survival curves were described. The univariable and multivariable Cox regression models were used to identify the co-variables that influence BCR. The software used to run the analysis was IBM-SPSS version 20. All tests were two-sided. *P <*0.05 was considered to be the threshold for statistically meaningful differences.

## Results

### Patients’ Clinicopathologic Characteristics and the Cutoff Values of SII and NLR for BCR

**Figure 1:**
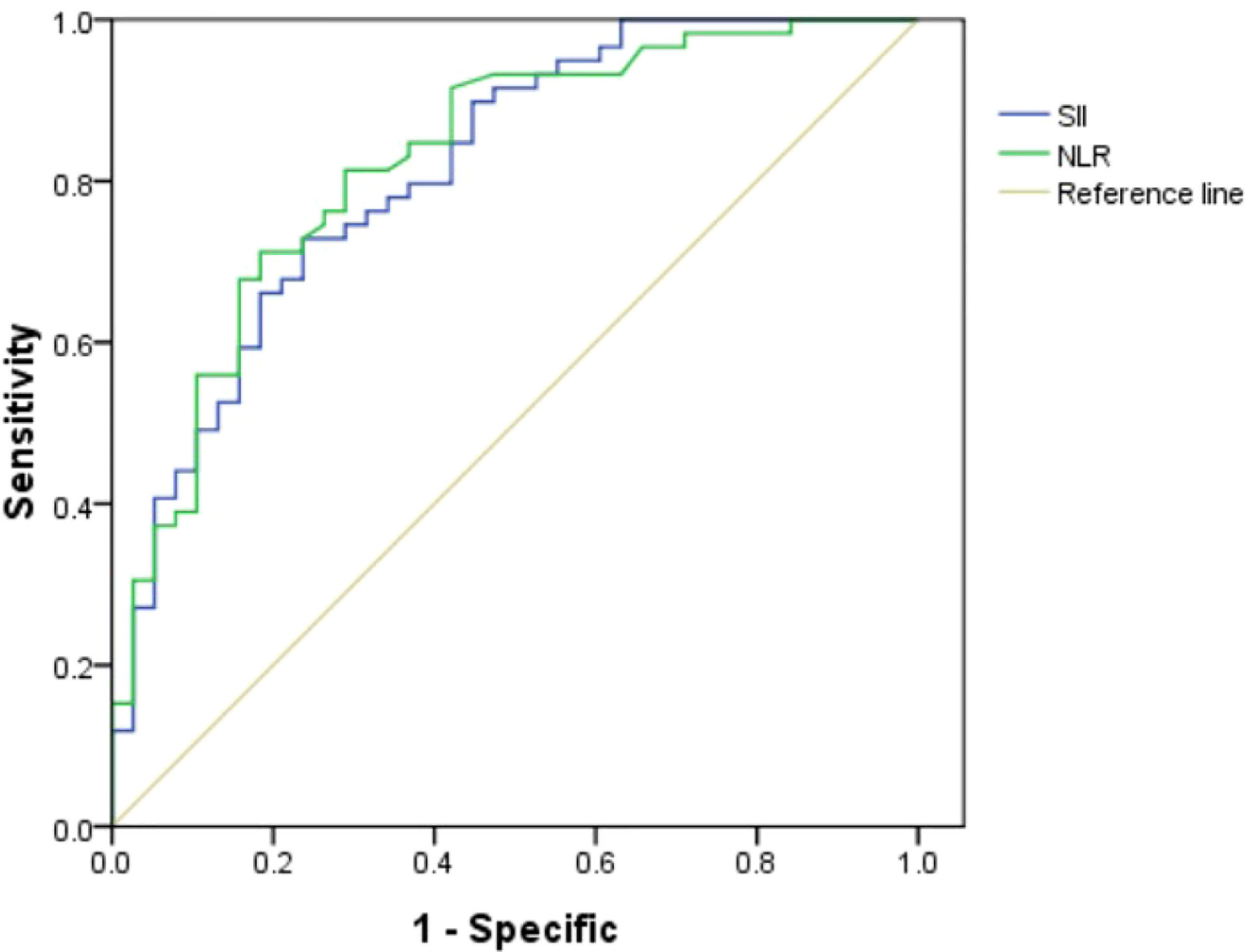
Role of the systemic immune-inflammation index (SII) and neutrophil–lymphocyte ratio (NLR) in predicting biochemical recurrence (BCR) after radical prostatectomy (RP) by ROC curve analysis. The AUC for NLR was 0.824 with *P*-value <0.001, and the AUC for SII was 0.813 with *P*-value <0.001.

A total of 291 patients with localized PCa were enrolled in the study. The median values of clinical factors were 66.13 ± 6.05 years for age, 24.35 ± 4.14 for BMI, 36.62 ± 23.15 ml for TPV, and 26.15 ± 33.77 ng/ml for tPSA. Twelve patients (4.12%) were pT1, 117 (40.21%) were pT2, and 162 (55.67%) were pT3. Thirty-nine patients (13.4%) were of low risk (GS ≤ 6), 129 (44.33%) were of intermediate risk (GS = 7), and 123 (42.27%) were of high risk (GS ≥ 8). Twenty patients (6.87%) were with pelvic lymph node metastases. The ROC of SII and NLR for BCR were analyzed to determine the optimal cutoff values for SII and NLR (**Figure 1**). The AUC for SII was 0.813, which was significantly lower than 0.05 (*P* < 0.001), with a threshold value of 528.54, a sensitivity of 72.9%, and a specificity of 76.3%; the AUC for NLR was 0.824, which was significantly lower than 0.05 (*P* < 0.001), with a threshold value of 2.62, a sensitivity of 71.2%, and a specificity of 81.6%; therefore, according to the threshold values of NLR and SII, the patients were divided into low-level and high-level groups, respectively. The patients’ clinicopathologic demographics are summarized in **Table 1**.

**Table 1:** Clinicopathological characteristics of the entire cohort and the NLR and SII subgroups.

	Mean (± SD) or counts (percentage of total)						
	Entire cohort	Low NLR	High NLR	*P*-value	Low SII	High SII	*P*-value
Number	291	141	150		129	162	
Age (years)	66.13 ± 6.05	64.79 ± 5.95	67.4 ± 5.92	0.68	65.24 ± 6.11	66.90 ± 5.95	0.72
BMI (kg/m^2^)	24.35 ± 4.14	25.11 ± 4.74	23.63 ± 3.38	0.08	24.78 ± 4.96	23.98 ± 3.29	0.77
tPSA (ng/ml)	26.15 ± 33.77	19.42 ± 21.14	32.74 ± 41.88	0.04	18.66 ± 18.49	32.89 ± 42.23	0.004
TPV (ml)	36.62 ± 23.15	35.31 ± 24.66	37.73 ± 21.96	0.62	35.84 ± 24.83	37.25 ± 21.90	0.71
GS (%)	291	141	150	0.02	141	150	0.04
≤6	39 (13.4)	21 (14.89)	18 (12.0)		24 (18.6)	15 (9.3)	
=7	129 (44.33)	72 (51.1)	57 (38.0)		57 (44.2)	72 (44.4)	
≥8	123 (42.27)	48 (34.0)	75 (50)		48 (37.2)	75 (46.3)	
pT stage (%)	291	141	150	0.006	129	162	0.007
pT1	12 (4.12)	9 (6.4)	3 (2)		4 (3.1)	8 (4.9)	
pT2	117 (40.21)	66 (46.8)	51 (34)		65 (50.4)	52 (32.1)	
pT3	162 (55.67)	66 (46.8)	96 (64)		60 (46.5)	102 (63)	

BMI, body mass index; TPV, total prostate volume; GS, Gleason score; pT, pathological stage; NLR, neutrophil–lymphocyte ratio; SII, systemic immune-inflammation index.

### Clinicopathological Characteristics in the Low and High NLR Groups

Initially, the distribution of clinicopathological characteristics was compared between groups according to the threshold value of NLR. The high NLR group showed unfavorable features compared with the low NLR group. In the high NLR group, preoperative serum tPSA (*P* = 0.04), GS (*P* = 0.02), and pT stage (*P* = 0.006) were significantly higher compared with those in the low NLR group, but the distribution of age, BMI, and TPV did not show any significant differences as shown in **Table 1**.

Then, univariable and multivariable logistic regression models were used to evaluate the association between NLR and several adverse pathological events. The results showed that there was no association between high NLR and pathological events including pT stage and GS as shown in **Tables 2****,** **3**.

**Table 2:** Univariable and multivariable analyses of the impact of NLR and SII on pathological T stage.

	Univariable analysis			Multivariable analysis		
	pT1–2a vs. pT2b–3			pT1–2a vs. pT2b–3		
	HR	95% CI	*P*-value	HR	95% CI	*P*-value
NLR < 2.62	1 (Ref)	1 (Ref)		1 (Ref)	1 (Ref)	
NLR ≥ 2.62	0.976	0.635–1.501	0.994	1.937	0.508–7.393	0.245
SII < 528.54	1 (Ref)	1 (Ref)		1 (Ref)	1 (Ref)	
SII ≥ 528.54	1.243	0.806–1.917	0.039	1.478	0.972–3.64	0.028

Ref, reference.

**Table 3:** Univariable and multivariable analyses of the impact of NLR and SII on GS.

	Univariable analysis			Multivariable analysis		
	GS ≤ 6 vs. GS ≥ 7			GS ≤ 6 vs. GS ≥ 7		
	HR	95% CI	*P*-value	HR	95% CI	*P*-value
NLR < 2.62	1 (Ref)	1 (Ref)		1 (Ref)	1 (Ref)	
NLR ≥ 2.62	1.393	0.857–2.263	0.172	1.327	0.810–2.176	0.245
SII < 528.54	1 (Ref)	1 (Ref)		1 (Ref)	1 (Ref)	
SII ≥ 528.54	1.577	0.965–1.578	0.038	1.656	1.00–2.742	0.042

Ref, reference.

### Clinicopathological Characteristics in the Low and High SII Groups

The distribution of clinicopathological characteristics was compared between groups according to the threshold value of SII. The high SII group showed unfavorable features compared with the low SII group. In the high SII group, preoperative serum tPSA (*P* = 0.004), GS (*P* = 0.04), and pT stage (*P* = 0.007) were significantly higher compared with those in the low SII group, but the distribution of age, BMI, and TPV did not show any significant differences as shown in **Table 1**.

Then, univariable and multivariable logistic regression models were used to evaluate the association between SII and adverse pathological events. In the univariable analysis, SII ≥528.54 was a risk factor associated with higher pT stage (HR, 1.243; 95% CI, 0.806–1.917, *P* = 0.039) and higher GS (HR, 1.577; 95% CI, 0.965–1.578, *P* = 0.038); in the multivariable analysis, SII ≥528.54 was an independent risk factor strongly associated with higher pT stage (HR, 1.478; 95% CI, 0.972–3.64, *P* = 0.028) and higher GS (HR, 1.656; 95% CI, 1.00–2.742, *P* = 0.042) as shown in **Tables 2****,** **3**.

### The Association Between NLR, SII, and BCR-Free Survival

**Figure 2:**
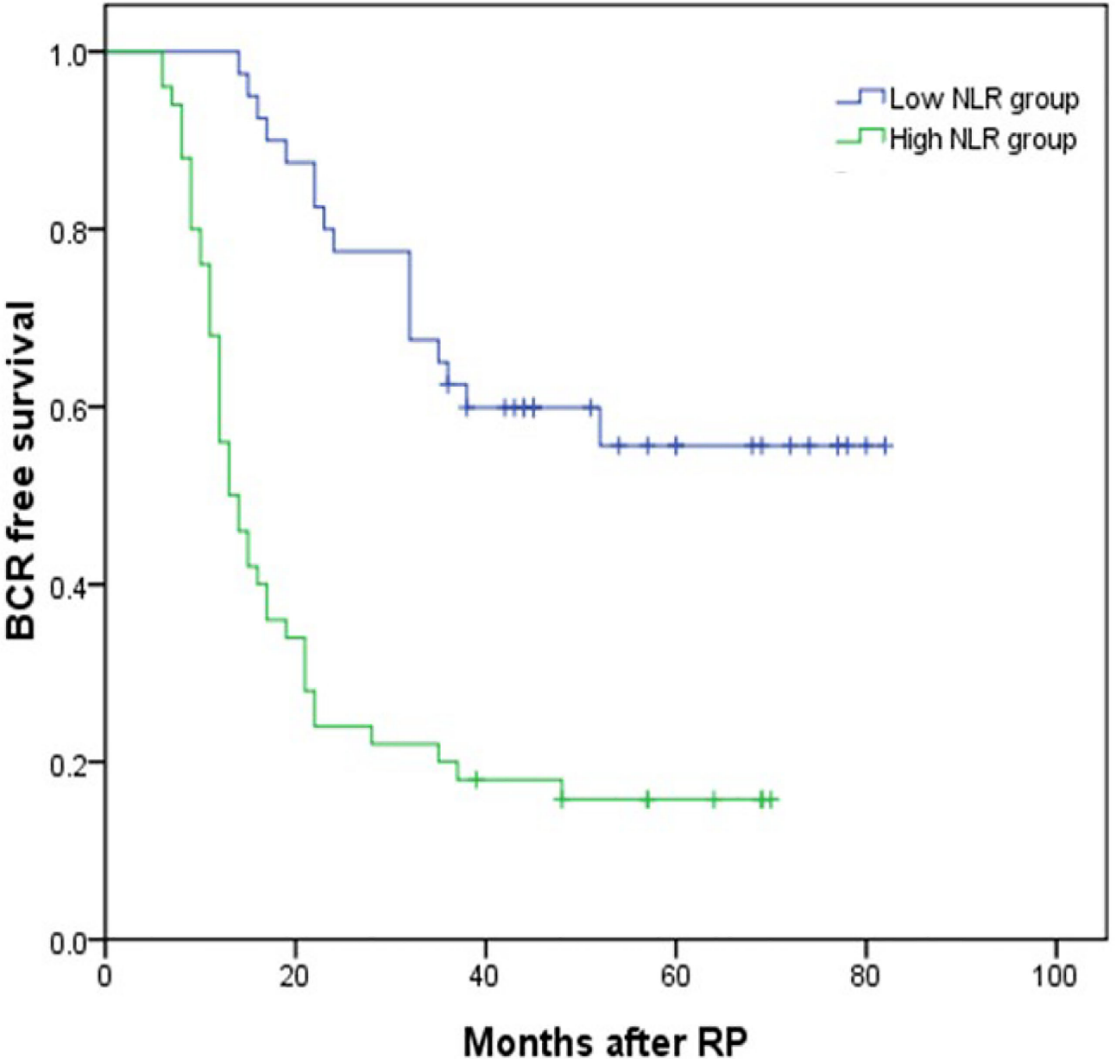
Kaplan–Meier curves for BCR-free survival according to NLR level. BCR-free survival of patients with NLR <2.62 was significantly longer than that of patients with NLR ≥2.62 (*P* < 0.001 by log-rank test).

The median follow-up time was 48 months (IQR 36–62) and 114 (39.18%) patients developed BCR. Thirty-eight (26.95%) and 76 (50.67%) patients developed BCR in the low and high NLR groups (*P* < 0.001). Kaplan–Meier analysis showed that BCR-free survival was significantly shorter in the high NLR group than in the low NLR group as shown in **Figure 2** (*P* < 0.001). By using the multivariable Cox regression model, it was revealed that NLR ≥2.62 (HR, 4.787; 95% CI, 2.339–9.798, *P* < 0.001) was a significant independent factor associated with BCR after RP as shown in **Table 4**.

**Table 4:** Cox regression analysis of potential factors associated with BCR after RP.

	Univariable analysis			Multivariable analysis		
	HR	95% CI	*P*-value	HR	95% CI	*P*-value
NLR < 2.62	1 (Ref)	1 (Ref)		1 (Ref)	1 (Ref)	
NLR ≥ 2.62	4.060	2.290–7.200	<0.001	4.787	2.339–9.798	<0.001
SII < 528.54	1 (Ref)	1 (Ref)		1 (Ref)	1 (Ref)	
SII ≥ 528.54	3.984	2.225–7.133	<0.001	4.521	2.262–9.037	<0.001
pT stage						
pT1	1 (Ref)	1 (Ref)		1 (Ref)	1 (Ref)	
pT2	1.633	0.217–12.279	1.633	5.667	0.661–48.622	0.114
pT3	2.998	0.812–21.864	<0.001	8.385	0.952–73.835	0.042
GS						
≤6	1 (Ref)	1 (Ref)		1 (Ref)	1 (Ref)	
=7	1.744	0.894–3.402	0.103	1.620	0.639–4.111	0.310
≥8	1.867	0.926–3.765	0.021	2.187	1.602–2.964	0.032

Ref, reference.

**Figure 3:**
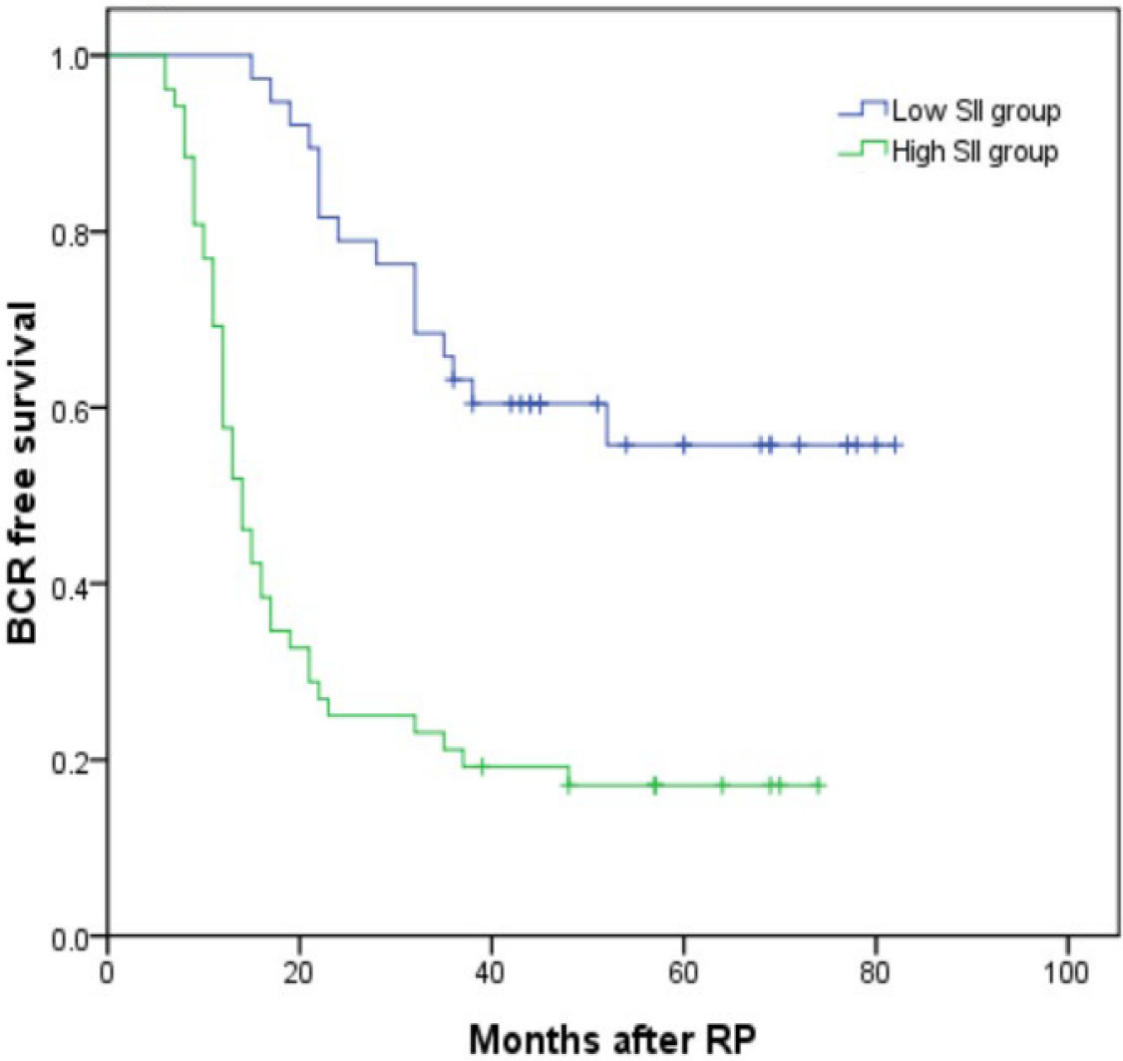
Kaplan–Meier curves for BCR-free survival according to SII level. BCR-free survival of patients with SII <528.54 was significantly longer than that of patients with SII ≥528.54 (*P* < 0.001 by log-rank test).

Thirty-five (27.13%) and 79 (48.77%) patients developed BCR in the low and high SII groups (*P* < 0.001). Kaplan–Meier analysis showed the BCR-free survival was significantly shorter in the high SII group than in the low SII group as shown in **Figure 3** (*P* < 0.001). By using the multivariable Cox regression model, it was revealed that SII ≥528.54 (HR, 4.521; 95% CI, 2.262–9.037, *P* < 0.001) was a significant independent factor associated with BCR after RP as shown in **Table 4**.

Meanwhile, the multivariable Cox regression model revealed that GS ≥8 (HR, 2.187; 95% CI, 1.602–2.964, *P* = 0.032) and pT3 stage (HR, 8.385; 95% CI, 0.952–73.835, *P* = 0.042) were also significant independent factors associated with BCR after RP as shown in **Table 4**.

## Discussion

In China, the incidence of PCa has been increasing in recent years. Among PCa patients after RP, BCR is one of the most important factors associated with the poor prognosis of patients (18). In a previous study, we investigated the association between NLR, SII, and the occurrence of PCa and revealed that high SII and NLR were all independent factors predicting PCa. SII seemed to be a more powerful tool compared with NLR (19). In this study, we further investigated the relationship between inflammatory factors and clinicopathological outcomes in localized PCa patients after RP and demonstrated that high SII and NLR were significantly associated with higher BCR rate and shorter BCR-free survival; meanwhile, high SII was strongly associated with higher GS and pT stage. To the best of our knowledge, this is the first study that investigated the relationship between SII and BCR in localized PCa patients after RP.

The relationship between inflammation and various kinds of malignant tumors has been reported by many studies (20, 21). The NLR based on the calculation of neutrophil to lymphocyte counts has been proposed as an indicator of general immune response to various stress stimuli and the host inflammatory status. An elevated NLR may be associated with both an increased neutrophil-dependent systemic inflammatory response and a lower lymphocyte-mediated antitumor immune response, reflecting a favorable immune microenvironment for tumor development and metastasis (22). In urological malignant tumors, inflammatory parameters have been considered important biomarkers for predicting bladder cancer progression (23). The relationship between NLR and clinical outcomes in PCa has been reported by many studies. Zhang et al. indicated that NLR ≥2.36 increased the risk of involvement of lymph nodes and was associated with higher GS (24). Another study revealed that NLR ≥2.5 was positively associated with GS, pT stage, and extracapsular extension (25). But only a few studies have investigated the role of NLR in predicting BCR after RP in localized PCa (26, 27). One study investigating the clinical outcomes in localized PCa patients after RP revealed that high NLR was significantly correlated with poor OS, CSS (cancer-specific survival), and BCR (27). Lee et al. demonstrated that NLR ≥2.5 was significantly related to unfavorable clinicopathological outcomes and worse BCR-free survival (25). Another study obtained the opposite conclusion: the study analyzed the data of 327 PCa patients who underwent robot-assisted RP and found that there was no correlation between NLR and PLR with BCR (28). However, the relationship between NLR and BCR remained controversial. In our study, we revealed that the threshold value of NLR for BCR was 2.62, which was similar to those reported by Lee et al. and Zhao et al. (25, 29). NLR ≥2.62 was significantly associated with poorer BCR-free survival according to Kaplan–Meier analysis and Cox regression analysis, but not associated with clinicopathological outcomes. We believed that NLR was an effective factor in predicting BCR in localized PCa patients after RP.

Recently, besides neutrophils and lymphocytes (30), the role of platelets has also been well-established in tumor occurrence and metastasis (31). SII, a novel inflammatory index that combines components of neutrophils, lymphocytes, and platelets, has been considered to reflect the systemic inflammatory responses more comprehensively than other inflammatory indexes. High SII suggested an elevated non-specific inflammatory status and a weak adaptive immune response in patients, which might promote the occurrence and progression of the tumor (32, 33). Several studies on inflammatory markers analyzed their predictive values in the PCa setting with various conclusions, but only a few of them included SII (34, 35). Our previous study demonstrated that high SII was an independent predictor for PCa, and it was one of the few studies detecting the role of SII in PCa (18). Recently, Rajwa et al. have evaluated the role of SII in non-metastatic PCa patients after RP and demonstrated that high preoperative SII ≥620 was independently associated with extracapsular extension, non-organ confined disease, and upgrading at RP (36). Another study evaluated the prognostic role of SII and NLR in mCRPC patients treated with abiraterone and revealed that SII ≥535 and NLR ≥3 were all independent predictors associated with shorter OS (12). Fan et al. obtained the same results and concluded that high SII could be used as a predictor for OS in mCRPC patients treated with abiraterone (37). However, none of these studies investigated the association between SII and BCR-free survival in localized PCa after RP. In our study, for the first time in the literature, the role of SII in predicting BCR-free survival was analyzed and the results indicated that high SII was significantly associated with shorter BCR-free survival. The cutoff value of SII for BCR was determined to be 528.54, and we also demonstrated that SII was associated with high BCR rate, pT stage, and GS, which was consistent with the conclusion of previous literature (36). For NLR, we failed to detect the association between NLR and pathological outcomes. Furthermore, both SII and NLR could represent the novel predictive markers for BCR in PCa patients after RP, and SII seemed more favorable for it was also associated with aggressive pathological outcomes.

This study still has some limitations. First, this was a single-center, retrospective study. Second, the biomarker was measured at a single time point, and it can be strengthened by collecting different preoperative sets of blood samples. Third, because of the relatively small sample size, more samples are needed in further studies.

## Conclusion

High preoperative SII was associated with higher GS and pT stage. High preoperative SII and NLR were related to higher BCR rate in localized PCa after RP, and they were all independent risk factors associated with shorter BCR-free survival. These two factors might provide promising and inexpensive methods predicting clinical outcomes in patients with RP. However, additional well-organized and large prospective studies are needed.

## Data Availability Statement

The raw data supporting the conclusions of this article will be made available by the authors, without undue reservation.

## Ethics Statement

This retrospective study was carried out at Peking University Cancer Hospital & Institute and got the approval of the Medical Ethics Review Committee of Peking University Cancer Hospital & Institute (protocol code 2020KT30).

## Author Contributions

PD, XS, and SW designed the study. SW, XY, ZY, YC, JM, PD, XS, XQY, and YY performed the study and analyzed the data. PD, XS, SW, XY, and ZY wrote the manuscript draft and revised the manuscript. All authors have read and agreed to the published version of the manuscript.

## Funding

This study was supported by the Capital’s Funds for Health Improvement and Research (2022-1G-1021) and the National Nature Science Foundation of China (82172604).

## Conflict of Interest

The authors declare that the research was conducted in the absence of any commercial or financial relationships that could be construed as a potential conflict of interest.

## Publisher’s Note

All claims expressed in this article are solely those of the authors and do not necessarily represent those of their affiliated organizations, or those of the publisher, the editors and the reviewers. Any product that may be evaluated in this article, or claim that may be made by its manufacturer, is not guaranteed or endorsed by the publisher.
